# Moral distress among healthcare professionals: Italian validation of the Moral Distress-Appraisal Scale

**DOI:** 10.3389/fpsyg.2026.1864410

**Published:** 2026-06-25

**Authors:** Marina Maffoni, Lidia Borghi, Giulia Lamiani

**Affiliations:** 1 Psychology Unit, Istituti Clinici Scientifici Maugeri IRCCS, Montescano Institute, Montescano, Italy; 2 Department of Health Sciences, University of Milan, Milan, Italy

**Keywords:** healthcare professionals, moral distress, professional ethics, psychometric properties, scale validation, subjective appraisal

## Abstract

**Introduction:**

Moral distress impacts healthcare professionals’ well-being, yet current measurement tools often rely on specific clinical scenarios, limiting their applicability. This study aimed to validate the Italian version of the Moral Distress-Appraisal Scale (MD-APPS), a context-independent instrument to assess the subjective appraisal of moral distress.

**Materials and methods:**

A cross-sectional validation study was conducted. 205 professionals caring for patients with oncological and chronic-degenerative diseases across different settings were enrolled. The sample was randomly split to perform Exploratory and Confirmatory Factor Analyses.

**Results:**

Participants reported a mean MD-APPS total score of 1.85 (SD = 0.76) (scale range 1–6). The analysis supported a robust two-factor structure consistent with the original framework: Support and Freedom (Factor 1), Hindrance and Coercion (Factor 2). The scale demonstrated satisfactory model fit indices [ $\chi^{2}(15)=11.0$ , p=0.756 ; SRMR = 0.039; RMSEA = 0.000 (90% CI: 0.000–0.067); CFI = 1.000; and TLI = 1.003] and adequate internal consistency (Factor 1, *α* = 0.82, *ω* = 0.83; Factor 2 *α* = 0.75, *ω* = 0.80; Total scale: *α* = 0.84; *ω* = 0.85). Convergent and divergent validity were substantiated through positive correlations with the Moral Distress Scale-Revised and the Burnout dimension of the Professional Quality of Life scale, and a negative correlation with Compassion Satisfaction dimension of the same scale.

**Discussion:**

The Italian MD-APPS appears a valid, reliable, and rapid-to-administer tool. By focusing on clinicians’ appraisals of moral distress, the MD-APPS is consistent with emerging definitions of moral distress (i.e., perceived violations of professional values or norms). Although further research is warranted, the MD-APPS holds promise as a suitable tool for diverse healthcare contexts and professional sectors.

## 1 Introduction

Since its first definition in 1984, moral distress has become a widely studied construct in healthcare research. Moral distress was originally defined as the suffering arising when a clinician “knows the right thing to do, but institutional constraints make it nearly impossible to pursue the right course of action” (Jameton, 1984). According to Jameton’s (1984) original definition, the core feature of moral distress was the experience of being constrained from acting in accordance with one’s professional values. Over the years, the literature has raised arguments aimed at refining and broadening Jameton’s (1984) definition.

Some authors have called for a revision of Jameton’s (1984) definition to include the subjective perception of being constrained and, accordingly, to recognize internal characteristics as potential sources of constraint (Hamric et al., 2012). In addition, other authors have highlighted that moral distress may occur in situations in which it is not necessarily clear what the “right” course of action is, but there is an intuition that something is wrong or not morally appropriate (Batho and Pitton, 2018). Despite these refinements, these authors did not challenge Jameton’s (1984) assumption that the core experience of moral distress lies in constraints on moral agency that prevent clinicians from acting in accordance with their professional values.

More recently, some authors have challenged the idea that moral distress is associated exclusively with moral constraint (Fourie, 2017) and have conceptualized moral distress as any form of psychological distress causally related to a moral event (Morley et al., 2019). According to this broader perspective, moral distress may arise from experiences of moral conflict, moral uncertainty, moral constraint, or moral dilemma (Morley et al., 2020). However, recent empirical evidence suggests that defining moral distress by focusing on its causes, as proposed by Morley et al. (2020), risks fragmenting the construct and overlooking its deeper, unifying dimension. In a recent qualitative study conducted among Italian healthcare professionals, a multi-layered model was proposed in which moral distress arises when different moral events entail a perceived violation of core professional values or norms (Lamiani et al., 2026).

Despite these conceptual differences, it is important to note that the most widely used instruments for assessing moral distress are based on a constraint-based definition of the construct (McCarthy and Gastmans, 2015). The main instruments developed to assess moral distress include the Moral Distress Scale (MDS) (Corley et al., 2001), the Moral Distress Scale–Revised (MDS-R) (Hamric et al., 2012), and the Measure of Moral Distress for Health Care Professionals (MMD-HP) (Epstein et al., 2019; Gorini et al., 2025). These scales, which have been validated in different countries, have been progressively adapted for use with different healthcare professionals. However, they assess moral distress by measuring the frequency and intensity of distress experienced by clinicians in response to a set of predefined morally distressing situations represented by the scale items. Consequently, these instruments primarily capture exposure to specific causes of moral distress rather than the individual’s appraisal of moral constraints and resources. At the same time, a recent review has shown that moral distress may arise from a wide range of organizational, interpersonal, and clinical factors that vary across healthcare settings (ShamsiKhani et al., 2025). Therefore, situations triggering moral distress may differ across contexts, and individuals may also differ in how they appraise and respond to such situations as morally distressing. To address this limitation, Baele and Fontaine (2021) developed the Moral Distress Appraisal Scale (MD-APPS), a context-independent instrument. The scale was subsequently validated in a Turkish sample (Duzgun et al., 2023), providing preliminary evidence of its cross-cultural applicability. Drawing on stress theories that emphasize the role of situational appraisal and perceived resources in the experience of stress, the MD-APPS assesses moral distress based on the subjective appraisal of the presence or absence of moral constraints in the work environment (e.g., “I am prevented from carrying out my work in a way that I believe is morally right”; “I can work in accordance with my norms and values”). Because it is context-independent and based on perceived constraints rather than objective exposure to predefined morally distressing situations, the MD-APPS can be used across different settings, professions and organizational cultures without the risk of underestimating the phenomenon. Despite these advantages, the MD-APPS has been validated only in Turkey (Duzgun et al., 2023) and has not yet been validated in Italy.

The aim of this study was to validate the Italian version of the MD-APPS in a sample of Italian healthcare professionals.

## 2 Materials and methods

### 2.1 Study design

This validation study was part of larger cross-sectional research on psychological well-being of healthcare professionals working with patients affected by oncology or chronic-degenerative diseases. In this validation study we explored three dimensions of construct validity of the MD-APPS: structural validity, convergent validity and divergent validity. Structural validity was examined through exploratory and confirmatory factor analyses. Convergent validity was assessed by examining the associations between the MD-APPS, the Italian version of the Moral Distress Scale-Revised (Lamiani et al., 2017), and burnout as measured by the Professional Quality of Life Scale, Version 5 (ProQuol-5) subscale (Stamm, 2009). Given that job satisfaction has been consistently found to be inversely related to moral distress, divergent validity was evaluated by examining the relationship between the MD-APPS and compassion satisfaction as measured by the ProQuol-5 subscale (Stamm, 2009).

### 2.2 Participants and procedures

All healthcare professionals caring for oncology patients or patients affected by chronic-degenerative diseases in different settings (hospital, long-term care facility, or home healthcare services) were eligible to participate. Other inclusion criteria were being able to understand written Italian language and having at least 1 year of experience in caring for these patients. Non-clinical staff was excluded from the study.

Participants were recruited by emails through institutional channels, including the Italian Association of Medical Oncology (AIOM), Italian Volunteers for Home Assistance to Suffering People (VIDAS), and the Italian League for the Fight against Cancer (LILT), as well as informal networks, such as direct email invitations to staff in oncology and palliative care units at three hospitals in northern and central Italy (ASST Santi Paolo and Carlo, Milan and Azienda Ospedaliero-Universitaria of Ferrara; ASL Toscana Centro) and one in southern Italy (ASL of Salerno). Data were collected through an online survey from May 2024 to January 2025.

### 2.3 Instruments

The survey included a brief section to collect socio-demographic data (gender, years of professional experience) and contextual data (work setting, availability of team debriefing, personal experience of illness) and three questionnaires: Moral Distress Appraisal Scale (MD-APPS) (Baele and Fontaine, 2021), the Italian Moral Distress Scale-Revised (MDS-R) (Lamiani et al., 2017) and Professional Quality of Life Scale, Version 5 (ProQuol-5) (Stamm, 2009).

The Moral Distress Appraisal Scale (MD-APPS) (Baele and Fontaine, 2021) is a self-report questionnaire recently developed to assess moral distress from a cognitive–appraisal perspective. Moral distress is conceptualized as the result of individuals’ evaluations of morally challenging situations and their perceived ability to cope with them. The MD-APPS consists of 8 items, rated on a six-point scale, from 1 ‘Totally disagree’ to 6 ‘Totally agree’, describing four key appraisals that imply the presence (‘hindrance’ and ‘coercion’) or absence (‘freedom’ and ‘support’) of moral constraint. The MD-APPS score is computed as the mean score of the hindrance and coercion facets and the reverse scores of the freedom and support facets (scale range 1–6). Higher scores at MD-APPS indicate higher levels of moral distress. For this validation study, the MD-APPS was translated into Italian following recognized guidelines for questionnaire adaptation (Sousa and Rojjanasrirat, 2011). Two bilingual Italian researchers initially translated the scale into Italian. The translation was then reviewed by an Italian language teacher to ensure linguistic accuracy and clarity. Subsequently, another bilingual English researcher performed a back-translation. The back-translated version and the original scale were compared by English- and Italian-speaking researchers to verify conceptual equivalence, semantic accuracy, and cultural appropriateness.

The Italian version (Lamiani et al., 2017) of the *Moral Distress Scale-Revised* (MDS-R) (Hamric et al., 2012), a self-report questionnaire designed to assess moral distress among healthcare professionals, defined as the psychological discomfort experienced when one is constrained from acting according to one’s moral or professional values. The MDS-R comprises 14 items, each describing a potentially morally distressing clinical situation. For each item, respondents are asked to rate the frequency with which the situation occurs, and the intensity of the moral distress experienced. Both dimensions are rated on a 5-point Likert scale ranging from 0 (*never/no distress*) to 4 (*very frequently / great distress*). Following standard scoring procedures, an item score is calculated by multiplying frequency by intensity. A total moral distress score is then obtained by summing the product scores across all items (scale range 0–16). Higher total scores indicate higher levels of moral distress.

The *Professional Quality of Life Scale*, Version 5 (ProQOL-5) (Stamm, 2009) consists of 30 items, rated on a 5-point Likert scale ranging from 1 (never) to 5 (very often), and is composed of three independent subscales, each including 10 items (subscale range 10-50). In this study we focused on the following two subscale: Compassion Satisfaction (CS), which reflects the pleasure and fulfilment derived from being able to do one’s work well and to help others; Burnout (BO), which captures feelings of emotional exhaustion, frustration, and hopelessness related to work demands. Subscale scores are calculated by summing the responses to the relevant items (after reverse scoring where appropriate), with higher scores indicating higher levels of the measured construct. The subscales are interpreted separately and are not combined into a total score. Higher scores on the Compassion Satisfaction subscale indicate greater professional fulfilment, whereas higher scores on the Burnout subscale indicate greater levels of fatigue and work-related distress.

### 2.4 Data analysis

Descriptive statistics were calculated to describe the sample characteristics and the scores obtained in study questionnaires. Means and standard deviations were reported for continuous variables, while frequency and percentages were used to summarize categorical data. To verify the homogeneity of the two subsamples (EFA and CFA), independent samples t-tests were conducted for continuous variables, and Pearson’s Chi-square tests were used for categorical variables.

To investigate the factorial structure of the MD-APPS, the overall sample was randomly split into two sub-samples. Exploratory Factor Analysis (EFA) was conducted on the first sub-sample. Prior to factor extraction, suitability for EFA was assessed by Bartlett’s test of sphericity (Bartlett, 1951) and the Kaiser–Meyer–Olkin (KMO) index (Kaiser, 1974), both the overall KMO value and individual item indices were required to surpass the generally accepted threshold of 0.6. EFA was performed using the Minimum Residuals extraction method and Oblimin rotation, which permits factor correlation. Conforming to the recommendations of (Finch, 2020), the number of factors retained was determined via parallel analysis in conjunction with scree plot inspection. Subsequently, item-factor loadings were analyzed to determine adequacy for model retention, adopting contemporary methodological standards (Howard, 2016): items were retained if their primary factor loading exceeded 0.40, loadings on alternative factors did not exceed 0.30, and the difference between primary and alternative loadings met or exceeded 0.20.

We also reported the explained variance of the factors. Generally, the explained variance of multifactorial scales is expected to exceed 40%, with higher values indicating stronger construct validity (Boateng et al., 2018).

Confirmatory Factor Analysis (CFA) was subsequently performed on the second sub-sample, employing structural equation modeling with the Diagonally Weighted Least Squares (DWLS) estimation method, which is suitable for ordinal data. Model fit was evaluated using conventional indices: Root Mean Square Error of Approximation (RMSEA), Comparative Fit Index (CFI), Tucker-Lewis Index (TLI), and Standardized Root Mean Square Residual (SRMR) (Cheung and Rensvold, 2002; Hu and Bentler, 1999; McDonald and Ho, 2002).

The goodness of fit was evaluated using the CFI (Bentler, 1990) and the TLI (Tucker and Lewis, 1973), with values >0.90 typically interpreted as indicating acceptable/good fit. We also considered the RMSEA (Steiger, 1990), for which values 〈0.08 suggest adequate fit and values 〈0.05 suggest good fit, as well as the SRMR (Jöreskog and Sörbom, 2002), with values 〈0.05 reflecting good fit and values between 0.05 and 0.07 indicating moderate fit. Residual covariances were added only when indicated by substantial modification indices and when justified by item content similarity, to account for local dependence without altering the theoretical structure of the model.

Convergent and divergent validity was assessed by estimating Pearson correlations between the total scores of the MD-APPS and those obtained from external, theoretically related scales, as well as their respective subdimensions. Specifically, because the ProQOL Burnout and Compassion Satisfaction subscales are inversely related but distinct dimensions of professional quality of life, Burnout was used as the convergent criterion, whereas Compassion Satisfaction was considered as an indicator of divergent/discriminant validity, particularly for Factor 2.

Positively worded “Freedom and Support” items were reverse-coded only to compute the total MD-APPS score (so that higher total scores indicate greater moral distress); factor scores were calculated and correlated using the original coding.

Internal consistency was evaluated via McDonald’s Omega (McDonald, 2013) and Cronbach’s Alpha (Cronbach and Meehl, 1955). Generally, internal reliability > 0.70 was considered acceptable (Cronbach and Meehl, 1955). Ninety-five percent confidence intervals were calculated to quantify the precision of the reliability estimates and to support their interpretation.

All statistical analyses were performed using Jamovi software version 2.6 and the R lavaan package (Navarro and Foxcroft, 2025). The significance threshold was established at *p* < 0.05 for all statistical tests.

### 2.5 Ethical statement

The study was approved by the Ethics Committee of the University of Salerno (Protocol Number 0064918 as of 19/02/2024), in compliance with the principles of the Declaration of Helsinki. Written informed consent was obtained from all participants, who were fully informed of the aims of the study and of the anonymity of the data collected. Participation in the study was voluntary, and no financial incentives were provided.

## 3 Results

### 3.1 Socio-demographic and occupational profile of the sample

A total of 205 healthcare professionals completed the survey. Participants had a mean age of 45.6 years (SD = 11.91), and an average length of professional service of 11.3 years (SD = 9.14). The sample was predominantly composed by female healthcare professionals (76.1%) working in hospital setting (48.8%). Regarding professional roles, physicians comprised 32.7% of the sample, nurses and healthcare assistants 27.8%, psychologists 19.5%, physiotherapists 7.3%, and volunteers 12.7%.

First, EFA was conducted on a randomly selected subsample (*n* = 102) of the total participant pool (*N* = 205). For analyses involving the EFA and CFA subsamples (*n* = 102 and *n* = 103, respectively), distributions of demographic and occupational characteristics closely matched those of the total sample (see Table 1).

**Table 1:** Socio-demographic and occupational profile of the sample.

Variable	Total sample (*N*. 205)	EFA sub-sample (*n*. 102)	CFA sub-sample (*n*. 103)	Statistic^a^ (*t*-test or *X*^2^)	*p*-value
	*Mean (SD)*				
Age	45.6 (11.91)	44.8 (11.77)	46.3 (12.07)	−0.888	0.376
Years of service	11.3 (9.14)	11.3 (8.88)	11.3 (9.43)	0.0351	0.972
	*N (%)*				
Gender					
Male	49 (23.9)	23 (22.5)	26 (25.2)	0.204	0.651
Female	156 (76.1)	79 (77.5)	77 (74.8)		
Profession					
Physician	67 (32.7)	38 (37.3)	29 (28.2)	7.630	0.106
Nurse/healthcare assistant	57 (27.8)	29 (28.4)	28 (27.2)		
Psychologist	40 (19.5)	17 (16.7)	23 (22.3)		
Physiotherapist	15 (7.3)	10 (9.8)	5 (4.9)		
Volunteer	26 (12.7)	8 (7.8)	18 (17.5)		
Geographic region					
North	124 (60.5)	57 (55.9)	67 (65.1)	2.900	0.407
Center	62 (30.2)	35 (34.3)	27 (26.2)		
South and Islands	15 (7.3)	7 (6.9)	8 (7.8)		
Missing	4 (2.0)	3 (2.9)	1 (1.0)		
Care setting					
Hospital	100 (48.8)	54 (52.9)	46 (44.7)	2.660	0.446
Home care	58 (28.3)	28 (27.5)	30 (29.1)		
Territorial services	34 (16.6)	13 (12.7)	21 (20.4)		
Private practice	13 (6.3)	7 (6.9)	6 (5.8)		
Disease of assisted patients					
Oncological	161 (78.5)	77 (75.5)	84 (81.6)	1.120	0.290
Chronic-progressive	44 (21.5)	25 (24.5)	19 (18.4)		
Age group of assisted patients					
Adult patients	186 (90.7)	92 (90.2)	94 (91.3)	0.069	0.792
Pediatric patients	19 (9.3)	10 (9.8)	9 (8.7)		
Presence of team supervision					
Yes	148 (72.2)	75 (73.5)	73 (70.9)	0.180	0.671
No	57 (27.8)	27 (26.5)	30 (29.1)		

a Independent samples *t*-tests were conducted for continuous variables, Pearson’s Chi-square tests were used for categorical variables.

### 3.2 Exploratory factor analysis

Data suitability for factor analysis was confirmed by Bartlett’s test of sphericity ( $\chi^{2}=521$ , df=28 , p<0.001), indicating that item correlations were adequate. The KMO measure yielded an overall value of 0.824, denoting good sampling adequacy; all individual KMO values exceeded the minimum acceptable threshold of 0.6.

**Figure 1:**
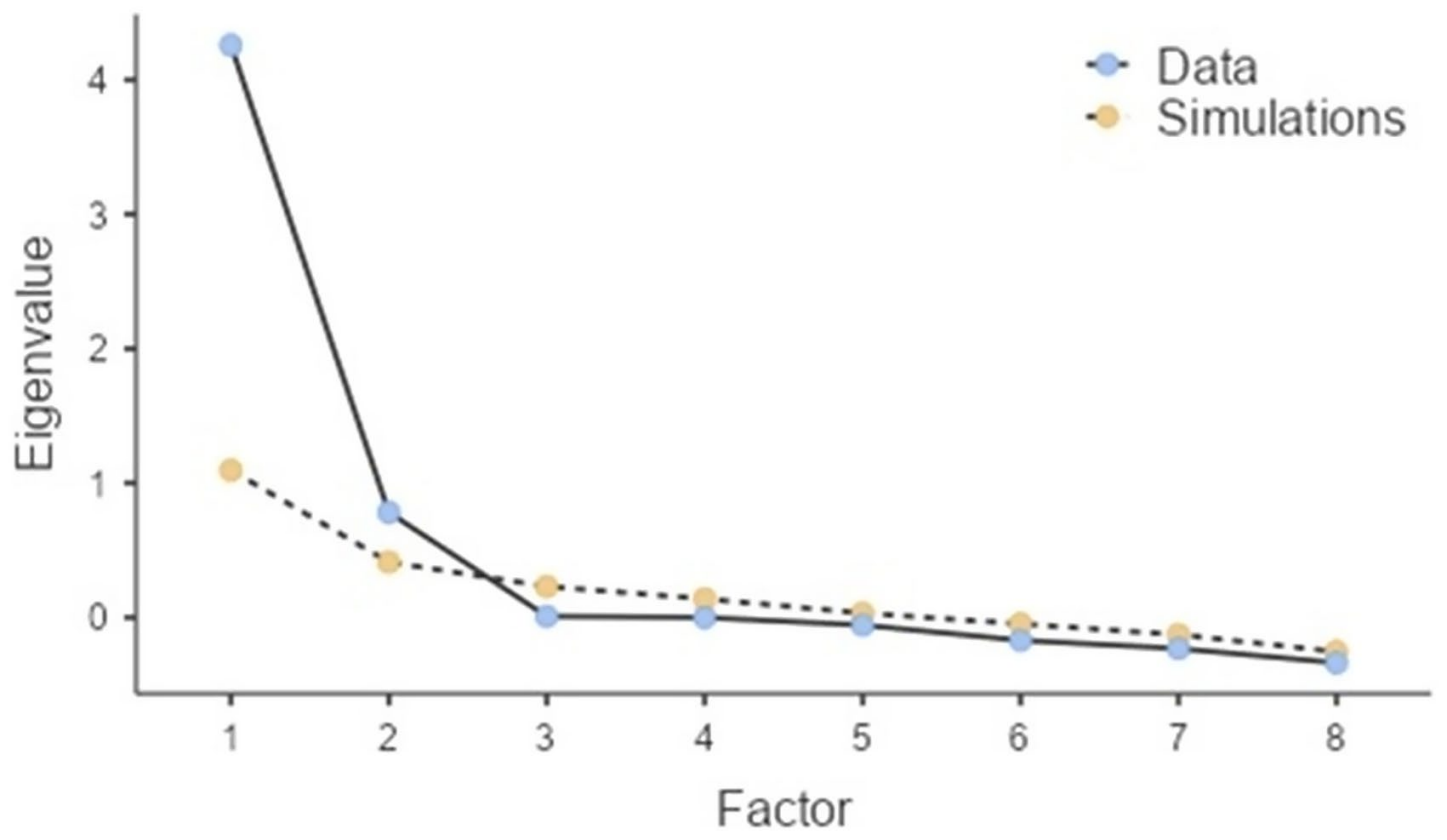
EFA scree plot.

Factors were extracted using the Minimum Residuals method. Subsequently, an Oblimin rotation was applied to allow for correlated factors. Item 7 showed a secondary loading on the other factor, which was retained as it remained below the threshold for problematic cross-loading and was theoretically compatible with the oblique rotation and the item content. Parallel analysis and inspection of the scree plot both supported the selection of a two-factor solution (see Figure 1).

Analysis of factor loadings revealed satisfactory saturation for all items, with loadings ranging from 0.563 to 0.977. Item uniqueness values were low, suggesting that the majority of item variance was explained by the latent factors (see Table 2). Overall, the two-factor model accounted for 67.1% of total variance, with Factor 1 (Support and Freedom) and Factor 2 (Hindrance and Coercion) explaining 33.8 and 33.3%, respectively.

**Table 2:** Factor loadings.

Questionnaire item	Factor		Uniqueness
	1	2	
	Support and Freedom	Hindrance and Coercion	
1. I am prevented from carrying out my work in a way that I believe is morally right [*Mi viene impedito di svolgere il mio lavoro in un modo che ritengo moralmente giusto*]		0.563	0.494
2. I can work in accordance with my norms and values. [*Riesco a lavorare in accordo con i miei principi e valori*]	0.627		0.426
3. I am required to do things that are contrary to my own norms and values [*Mi viene richiesto di fare cose che sono contrarie ai miei principi e valori*]		0.774	0.426
4. I am helped to work in a way that I believe is morally right [*Sono aiutato a lavorare in un modo che ritengo moralmente giusto*]	0.977		0.135
5. I can work in a way that I believe is morally right. [*Riesco a lavorare in un modo che ritengo moralmente giusto*]	0.681		0.270
6. I am compelled to do things that I believe are morally wrong [*Sono costretto a fare cose che ritengo moralmente sbagliate*]		0.878	0.253
7. I am supported to act ethically [*Sono supportato ad agire eticamente*]^a^	0.822		0.347
8. I am kept from working ethically [*Mi viene impedito di lavorare eticamente*]		0.835	0.279

a Factor extraction was performed using the Minimum Residuals method with Oblimin rotation. Item 7 showed a secondary loading on the second factor <0.20, consistent with the oblique rotation applied.

### 3.3 Confirmatory factor analysis

CFA was performed on a second sub-sample (*n* = 103), using structural equation modeling with the Diagonally Weighted Least Squares (DWLS) estimation method. The hypothesized model confirmed two latent factors, as identified by the EFA.

The first factor, labelled as in original scale, *Support and Freedom* (item 2, item 4, item 5, item 7), reflects the extent to which individuals perceive alignment between their work practices and their personal ethical standards. This dimension captures experiences of moral congruence and institutional support for ethical behavior. Items loading on this factor emphasize the ability to act in accordance with one’s values, to perform tasks perceived as morally right, and to receive organizational or collegial reinforcement in doing so. High scores on this factor represent a sense of coherence between personal moral values and professional actions.

The second factor, labelled as in original scale, *Hindrance and Coercion* (item 1, item 3, item 6, item 8), represents the perceived barriers that hinder individuals from acting in accordance with their moral values and ethical standards. The items composing this factor capture experiences of external or institutional pressures that limit one’s ability to behave ethically. High scores on this factor indicate greater perceived obstruction in carrying out morally appropriate actions, reflecting situations in which professionals feel constrained or coerced into actions that conflict with their moral beliefs.

Covariances between the error terms of selected items (item 4 ~~ item 7; item 2 ~~ item 5; item 2 ~~ item 7; item 7 ~~ item 6) were incorporated because the corresponding modification indices were substantial and the item pairs showed clear conceptual redundancy or near-synonymous wording, which plausibly generated shared residual variance beyond the latent factors. Specifically, item 4 and item 7 both refer to external support for ethical action; item 2 and item 5 both refer to freedom to act according to one’s values; item 2 and item 7 capture closely related aspects of Support and Freedom in moral action; and item 7 and item 6 reflect adjacent, conceptually linked poles of the moral-agency domain. These covariances were introduced to account for local dependence and shared wording, rather than to achieve a purely data-driven improvement in fit.

Model fit indices indicated excellent fit: $\chi_{\mathrm{df}=15}^{2}=11.0$ , p=0.756 ; SRMR = 0.039; RMSEA = 0.000 (90% CI: 0.000–0.067); CFI = 1.000; and TLI = 1.003 (see Table 3).

**Table 3:** CFA Fit indices of the two-factors model.

Type	SRMR	RMSEA	95% Confidence Intervals		RMSEA p
			Lower	Upper	
Classical	0.039	0.000	0.000	0.067	0.896
Robust	0.035				
Scaled	0.035	0.092	0.034	0.144	0.098

Model fit indices	Model
Comparative Fit Index (CFI)	1.000
Tucker–Lewis Index (TLI)	1.003

### 3.4 Reliability of MD-APPS

The internal reliability of the MD-APPS subscales was explored by both Cronbach’s alpha and McDonald’s omega coefficients. Specifically, MD-APPS Factor 1 demonstrated strong internal consistency (*α* = 0.82, 95% CI = 0.76–0.87; *ω* = 0.83, 95% CI = 0.79–0.86), while MD-APPS Factor 2 showed acceptable reliability (*α* = 0.75, 95% CI = 0.66–0.82; ω = 0.80, 95% CI = 0.75–0.84). For the total MD-APPS scale, both indices indicated excellent reliability (α = 0.84, 95% CI = 0.78–0.88; ω = 0.85, 95% CI = 0.81–0.88).

### 3.5 Convergent and divergent validity

The MD-APPS factors demonstrated inter-correlations and meaningful associations with related constructs, supporting convergent validity (i.e., MDS-R and ProQOL burnout) and divergent validity (i.e., ProQOL compassion satisfaction). Given that ProQOL Burnout and Compassion Satisfaction are inversely related subscales ( r=−0.639 ), Burnout was used as the convergent criterion, whereas Compassion Satisfaction was considered in the divergent/discriminant validity analysis, particularly for Factor 2.

As attended MD-APPS Factor 1 Support and Freedom correlated negatively with MD-APPS Factor 2 Hindrance and Coercion (*r* = −0.52, *p* < 0.001) and with all the MDS-R subscales (e.g., *r* = −0.20 to −0.38, *p* ranging from <0.05 to < 0.001), except for MDS-R Communication. MD-APPS Factor 2 Hindrance and Coercion correlated positively with MDS-R subscales (e.g., *r* = 0.28 to 0.40, *p* from <0.01 to <0.001). The total MDS-R score correlated moderately with both MD-APPS factors (Support and Freedom: *r* = −0.31, *p* < 0.01; Hindrance and Coercion: *r* = 0.43, *p* < 0.001). Similarly, the total score of the two scales correlates moderately (*r* = 0.42, *p* < 0.001) (see Table 4).

**Table 4:** Correlation between MD-APPS and MDSR.

Moral distress scales	Mean	SD	1	2	3	4	5	6	7	8
1. MD-APPS Factor 1 (Support and Freedom)	2.13	0.925	—							
2. MD-APPS Factor 2 (Hindrance and Coercion)	1.57	0.824	−0.522^***^	—						
3. MDS-R futile care	4.20	3.379	−0.204^*^	0.324^***^	—					
4. MDS-R misconduct	2.48	2.207	−0.252^*^	0.397^***^	0.656^***^	—				
5. MDS-R communication	4.85	3.402	−0.170	0.281^**^	0.575^***^	0.660^***^	—			
6. MDS-R teamwork	4.43	3.641	−0.377^***^	0.376^***^	0.389^***^	0.556^***^	0.469^***^	—		
7. MDS-R total score	3.78	0.925	−0.311^**^	0.425^***^	0.798^***^	0.885^***^	0.825^***^	0.748^***^	—	
8. MD-APPS total score	1.85	0.763	−0.888^***^	0.856^***^	0.298^**^	0.367^***^	0.255^**^	0.431^***^	0.418^***^	—

^*^p < 0.05, ^**^p < 0.01, ^***^p < 0.001.

As shown in Table 5, MD-APPS Factor 1 Support and Freedom showed a significant positive correlation with ProQOL compassion satisfaction (*r* = 0.26, *p* < 0.01) and a significant negative correlation with ProQOL burnout (*r* = −0.38, *p* < 0.001). MD-APPS Factor 2 Hindrance and Coercion showed weaker correlation with ProQOL burnout (*r* = 0.26, *p* < 0.01). The total MD-APPS scale positively correlates only with ProQOL burnout (*r* = 0.37, *p* < 0.001).

**Table 5:** Correlation between MD-APPS and ProQOL.

Scales	Mean	SD	1	2	3	4	5
1. MD-APPS Factor 1 (Support and Freedom)	2.13	0.925	—				
2. MD-APPS Factor 2 (Hindrance and Coercion)	1.57	0.824	−0.522^***^	—			
3. MD-APPS total score	1.85	0.763	−0.888^***^	0.856^***^	—		
4. ProQOL compassion satisfaction	39.24	5.46	0.260^**^	0.022	−0.146	—	
5. ProQOL burnout	22.09	5.85	−0.378^***^	0.263^**^	0.371^***^	−0.639^***^	—

^*^p < 0.05, ^**^p < 0.01, ^***^p < 0.001.

## 4 Discussion

The primary aim of this study was to validate the Italian version of the MD-APPS and to assess its psychometric properties among a sample of healthcare professionals working with patients affected by oncology or chronic-degenerative diseases. The findings confirm that the Italian version of the MD-APPS is a reliable and valid instrument for measuring moral distress, demonstrating a stable internal structure and satisfactory convergent and divergent validity.

Regarding the psychometric structure, the EFA and CFA yielded a two-factor solution, identifying “Support and Freedom” (Factor 1) and “Hindrance and Coercion” (Factor 2) as distinct dimensions of the construct. This internal structure is fully consistent with the original scale developed by Baele and Fontaine (2021), which conceptualized moral distress through the appraisal of resources (moral congruence) and stressors (moral constraints). Furthermore, the replication of this two-factor structure aligns with the recent Turkish validation by Duzgun et al. (2023), supporting the cross-cultural stability of the instrument. Internal consistency was adequate, with Cronbach’s alpha values ranging from 0.75 to 0.84, accompanied by 95% confidence intervals, and supported by McDonald’s omega. The confidence intervals further indicate that these estimates were reasonably precise and stable, suggesting that the observed reliability is unlikely to reflect substantial sampling fluctuation. This suggests that the Italian MD-APPS accurately captures the theoretical components of moral distress as originally intended.

Regarding convergent validity, the analysis revealed moderate correlations between the MDS-R and the MD-APPS (Factor 1 Support and Freedom, *r* = −0.31; Factor 2 Hindrance and Coercion, *r* = 0.43; total scale, *r* = 0.42). This moderate magnitude likely reflects the distinct structural and theoretical foundations of the two instruments. While the MDS-R relies on a checklist of specific, context-dependent clinical scenarios, the MD-APPS is designed as a context-independent tool focused on the *subjective appraisal* of moral constraints rather than situational frequency. This difference is theoretically meaningful: recent qualitative evidence suggests that moral distress is not defined by the objective presence of specific morally troubling situations or causes, but by the professional’s appraisal that such situations entail a violation of professional values or norms (Lamiani et al., 2026). Despite this difference in measurement approach, the direction of the association is entirely consistent with theoretical expectations, confirming that the MD-APPS validly captures the core construct of moral distress while offering a broader, cross-situational perspective. A similar pattern emerged regarding the correlations with the ProQOL dimensions. The negative association with burnout (Factor 1 Support and Freedom, *r* = −0.38; Factor 2 Hindrance and Coercion, *r* = 0.26) and the negative association with compassion satisfaction (Factor 1 Support and Freedom, *r* = −0.26) were statistically significant and in the expected direction, supporting the construct validity of the MD-APPS. Of note, MD-APPS Factor 2 (Hindrance and Coercion) showed no meaningful association with ProQOL Compassion Satisfaction ( r=0.022 ), further supporting discriminant validity by indicating that perceived hindrance and coercion are distinct from the positive sense of fulfillment captured by Compassion Satisfaction. Considering the overall scale, MD-APPS correlates only with burnout (*r* = 0.37). However, the magnitude of these correlations was moderate, suggesting that while moral distress is a contributing factor to professional quality of life, it remains a distinct construct. This finding is consistent with literature on moral distress that define moral distress as a specific form of stress rooted in perceived threats to professional values and congruence, rather than as a general indicator of emotional exhaustion or occupational strain. The MD-APPS specifically targets the appraisal of moral congruence and constraints, whereas burnout and compassion satisfaction represent broader emotional and psychological outcomes of helping professions. This distinction reinforces the utility of the MD-APPS as a specific measure of moral stress experience rather than a general indicator of occupational well-being.

### 4.1 Strengths and limits

A major strength of the MD-APPS lies in its unique theoretical and methodological approach compared to existing tools currently used in Italy and internationally, such as MDS (Corley et al., 2001), MDS-R (Hamric et al., 2012; Lamiani et al., 2017), and MMD-HP (Epstein et al., 2019; Gorini et al., 2025). While these instruments rely on a list of predefined, context-specific situations (e.g., specific clinical scenarios), the MD-APPS is context-independent. This feature is particularly relevant considering recent evidence showing that moral distress arises across heterogeneous contexts and is caused by the perceived violation of professional values or norms (Lamiani et al., 2026). This feature represents a significant advancement, as it may facilitate the assessment of moral distress beyond specific healthcare contexts. Emerging research has documented the presence of moral distress in diverse professional sectors, including social work, education, law enforcement, and business settings (Bernhardt et al., 2021; Mänttäri-van Der Kuip et al., 2024; Sugrue, 2020; Papazoglou and Chopko, 2017; Nielsen et al., 2024; Prottas, 2013). However, the present study was conducted exclusively among healthcare professionals working with oncology and chronic-degenerative patients. Therefore, although the appraisal-based structure of the MD-APPS may theoretically support its use across occupational sectors, further research is needed before broader applicability can be assumed. Future studies should examine the factorial validity, measurement invariance, and psychometric performance of the MD-APPS in non-healthcare professions before claims regarding cross-sector applicability can be made. Rather than anchoring moral distress to a fixed set of morally distressing events, the MD-APPS operationalizes the construct at a deeper level, focusing on the appraisal of threats to professional moral integrity that cut across professions and organizational contexts. Thus, the MD-APPS is not anchored to specific, *a priori* defined experiences that may become obsolete or irrelevant in different settings. Instead, it is grounded in a theoretical framework of stress that emphasizes *subjective appraisal*. By focusing on the individual’s perception of constraint rather than the objective presence of external barriers, the scale captures the transversal, subjective nature of moral distress. This approach avoids the pretension of objectivity regarding external constraints, acknowledging that the experience of distress is fundamentally mediated by how individuals perceive their ability to act in accordance with their values.

An additional practical strength of the Italian MD-APPS is its brevity, which makes it rapid to. Given that the instrument comprises a limited number of items, it can be easily integrated into routine assessments or larger survey batteries without substantially increasing respondent burden, an aspect that may facilitate its use in time-constrained workplaces.

Although these positive facets, several limitations should be acknowledged. First, while the scale is theoretically designed to be context-independent, the sample used for this validation—though differentiating between professions—was restricted to the disciplinary sector of chronicity (e.g., oncology and palliative care). Consequently, the findings may not fully generalize to professionals working in acute care, rehabilitation, or other high-intensity clinical settings where the nature of moral stressors might differ. Future studies should examine whether the appraisal-based structure of the MD-APPS remains stable across settings characterized by different organizational pressures and ethical climates.

Second, the total sample size was relatively small (*N* = 205), compared with original (*N* = 406) and Turkish (*N* = 420) validations, which may limit generalizability. Moreover, the sample was randomly split into two groups for conducting EFA and CFA, which may limit the generalizability and stability of the factor structure. Indeed, small sample sizes can lead to increased uncertainty in parameter estimates and may reduce the precision of model fit indices. Of note, the two randomly generated subsamples did not differ significantly with respect to professional roles or key demographic characteristics as shown in Table 1. Additionally, certain error covariances were introduced in the CFA to improve model fit, indicating possible residual shared variance among some items, potentially due to linguistic or semantic similarities. This suggests some item responses may not be fully independent, which can impact discriminant validity.

Third, the study did not assess test–retest reliability or measurement invariance across subgroups, limiting understanding of the scale’s stability over time and applicability across different populations. Finally, although the translation process followed established guidelines, subtle cultural nuances might not be fully captured, and further qualitative validation could enhance the cross-cultural appropriateness of the instrument.

## 5 Conclusion

This study validates the Italian version of the MD-APPS as a reliable and valid tool for assessing moral distress. The confirmed two-factor structure (*Support and Freedom* and *Hindrance and Coercion*) and its context-independent design allow for a subjective appraisal of moral stressors beyond specific clinical scenarios. Brief and easy to administer, the MD-APPS is a promising instrument for both research and practice, offering a standardized metric to investigate moral distress across diverse professional settings and to inform targeted interventions for workers well-being.

## Data Availability

The raw data supporting the conclusions of this article will be made available by the authors, without undue reservation.
